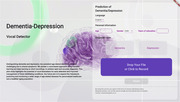# AI‐based dementia risk prediction using voice digial biomarkers: A web‐based platform

**DOI:** 10.1002/alz.088519

**Published:** 2025-01-09

**Authors:** Hyunwoong Ko, Nayoung Park, Sukbong Kwon

**Affiliations:** ^1^ MAGO inc., Seoul Korea, Republic of (South)

## Abstract

**Background:**

Dementia is a major public health problem affecting millions of people worldwide. Early diagnosis and intervention are essential to improve quality of life and reduce the burden of dementia. Recently, voice digital biomarkers have emerged as a promising approach for the early detection of dementia owing to its clinical utility and accessibility.

**Method:**

In this study, we present a voice biomarker‐based dementia prediction system using a Korean dataset from the AI hub entitled cognitive disorder diagnostic speech/dialogue. A total of 5,769 voice recordings were obtained, of which 2,424 were from CN, 1,611 from MCI, and 1,734 from AD, and were recorded during the administration of cognitive tests. We first extracted dementia‐related speech biomarkers using signal processing and speech activity detection. The extracted speech biomarkers were then used to predict the Clinical Dementia Rating (CDR) to discriminate the severity of dementia from the dataset using ML/DL framework. We also developed an automated engine to extract the voice biomarkers and predict the CDR. The engine was deployed using Docker containers and a web service was implemented using the Flutter framework.

**Result:**

The results showed that the proposed system achieved a CDR prediction accuracy of 83.6% and precision of 0.872. This is comparable to the accuracy of other voice biomarker‐based dementia prediction systems developed using Western datasets. A Flutter‐based web service is shown in the attached figure.

**Conclusion:**

The proposed system is a promising new approach for early detection of dementia. It is easy to use, accessible and inexpensive, making it a potential tool for widespread use. The system could be used to screen large numbers of people for dementia, potentially leading to earlier diagnosis and intervention. In addition, the use of a low‐language digital voice biomarker makes the system more accessible to people from different linguistic backgrounds. This could help to reduce inequalities in dementia detection and care. In the future, we plan to add a variety of predictive models, including depression and Parkinson's disease. More detailed information, including a published web site, will be released through the AAIC 2024 poster session.